# Structural and molecular basis for foot-and-mouth disease virus neutralization by two potent protective antibodies

**DOI:** 10.1007/s13238-021-00828-9

**Published:** 2021-02-18

**Authors:** Hu Dong, Pan Liu, Manyuan Bai, Kang Wang, Rui Feng, Dandan Zhu, Yao Sun, Suyu Mu, Haozhou Li, Michiel Harmsen, Shiqi Sun, Xiangxi Wang, Huichen Guo

**Affiliations:** 1 State Key Laboratory of Veterinary Etiological Biology and National Foot and Mouth Disease Reference Laboratory, Lanzhou Veterinary Research Institute, Chinese Academy of Agricultural Sciences, Lanzhou, 730046 China; 2 CAS Key Laboratory of Infection and Immunity, CAS Center for Excellence in Biomacromolecules, Institute of Biophysics, Chinese Academy of Sciences, Beijing, 100101 China; 3 University of Chinese Academy of Sciences, Beijing, 100049 China; 4 Division Virology, Wageningen Bioveterinary Research, P.O. Box 65, 8200 AB Lelystad, The Netherlands

**Dear Editor,**

Foot-and-mouth disease (FMD) is an economically devastating and highly contagious viral disease of cloven-hoofed animals with a global distribution. The causative agent, FMD virus (FMDV) is a small non-enveloped RNA virus, belonging to the *Aphthoviruses* genus within *Picornaviridae* family (Tuthill et al., 2010). Control of FMD has been largely reliant on vaccinations with inactivated virus vaccines. However, significant antigenic diversity within FMDV serotypes and inability of the vaccines to induce immune protection for a long duration of time impinge on the efficacy of available vaccines. The roles of neutralizing antibodies (NAbs) as the principal protective components of the immune responses to FMDV vaccination or infection have been well established (Pay and Hingley, 1987; Juleff et al., 2009). Passive immunization of NAbs has also been demonstrated to be effective in curing FMD and many viral diseases (Harmsen et al., 2007; Qiu et al., 2018). A deep understanding of the molecular basis for viral neutralization by antibodies and the identification of key viral epitopes would aid in the development of potent rationally designed broad-spectrum vaccine.

By using phage display immune libraries derived from four llamas, we previously identified 24 single-domain antibodies capable of neutralizing FMDV type O *in vitro* (Harmsen et al., 2007). Among these, M8 and M170 exhibited relatively strong neutralizing activities. To investigate the serotype specificity or cross-reactivity of M8 and M170, we propagated and purified FMDV O, A, Asia 1 as well as C serotype strains and separately examined their binding abilities to each antibody by enzyme-linked immunosorbent assay (ELISA). The ELISA results revealed that M170 only binds to type O, but M8 is capable of reacting with all the indicated serotypes, suggesting that M8 and M170 are type cross-reactive and O-specific, respectively (Fig. S1A). Surface plasmon resonance (SPR) experiments verified that both M8 and M170 interact with type O with a high binding affinity of 0.5 nmol/L and 1.0 nmol/L, respectively (Fig. 1A). To explore whether these two antibodies can simultaneously bind the virus, we performed a competitive SPR assay and the results indicated that the binding of one antibody blocks the attachment of the other (Fig. 1B), which suggests that M8 and M170 compete with each other for simultaneous binding albeit with different characteristics in binding distinct serotypes and targeting distinct antigenic sites. Cell-based neutralization assays showed that both M8 and M170 exhibit potent neutralizing activities against type O with a 50% neutralizing concentration value (Neut_50_) of 0.8 and 3.2 μmol/L, respectively (Fig. 1C, up). Perhaps correlated with the inability of simultaneous binding, the cocktail of M8 and M170 did not exhibit synergistic neutralization activity (Fig. 1C, down). Interestingly, results of a fluorescence-based assay revealed that M8, rather than M170, destabilized FMDV particles by 3–8 °C in an incubation time dependent manner at physiological conditions (pH 7.5), indicating that physical destabilization of viral particles that interferes with normal uncoating may be a possible neutralization mechanism for M8 (Fig. S1B).

**Figure 1:**
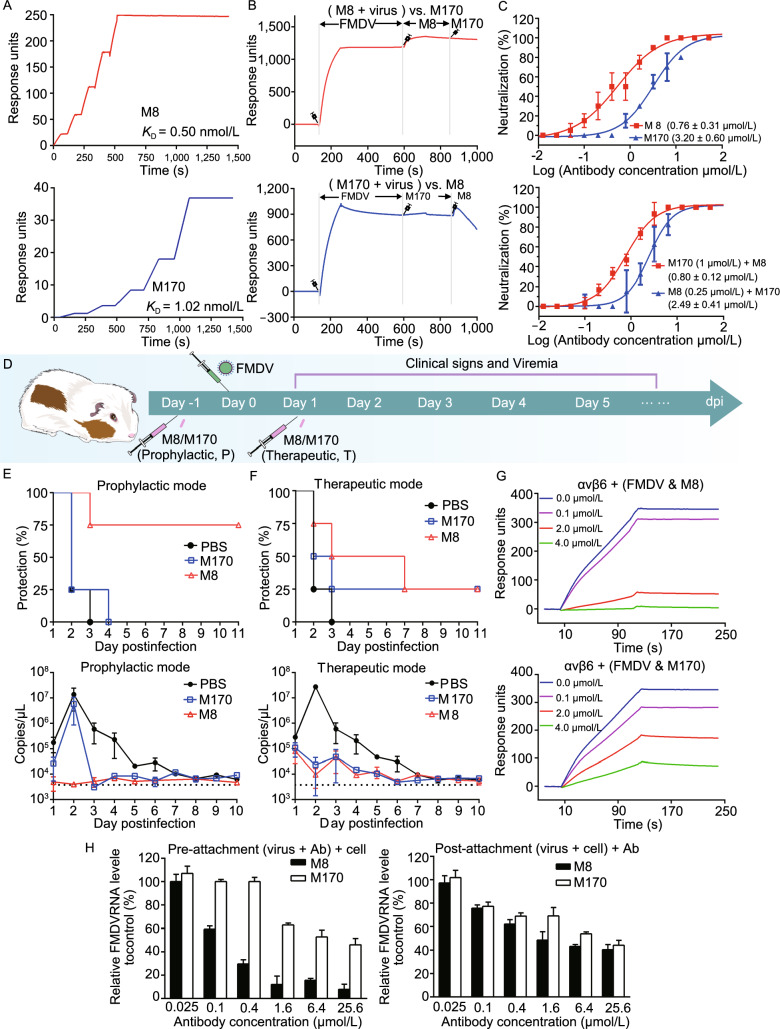
**Characterizations of anti-FMDV NAbs M8 and M170**. (A) BIAcore SPR kinetic profile of mAb M8 (top) and M170 (bottom) against FMDV O. The binding affinity is depicted by *K*_D_ (equilibrium dissociation constant, *K*_D_ = *K*_d_/*K*_a_), which was calculated by the BIAcore 3000 analysis software (BIAevaluation version 4.1). (B) Analysis of the simultaneous binding of M8 and M170 to FMDV through a competitive SPR assay. Due to the requirement of acidic condition for the sensor-labeling, M8 or M170, rather than FMDV, was loaded onto the sensor. In the upper panel, the sensor was labeled with M8. FMDV was inject first, followed by the second injection of M8 again to fully occupy all binding sites for M8 on viral surface, then M170 flowed through. In the bottom panel, the sensor was labeled with M170. The SPR procedure of M170 is same to that of M8. Note: signals (during 800–1000 s) decreased upon M8 binding in the bottom panel, which might result from the ability that M8 has the potential to strip FMDV off the M170-labelled sensor due to the higher binding affinity. (C) Neutralization of FMDV O by M8/M170 (top) and the combination of M8 and M170 (bottom) using plaque-reduction neutralization assay. Neut_50_ values indicate concentration of antibody required to neutralize fifty percent of the viral titer. The Neut_50_ of M8 and M170 were 0.8 μmol/L and 3.2 μmol/L, respectively. The Data is presented as the mean ± SD of triplicate measurements. (D) Schematic diagram of M8 or M170 treatment in guinea pig model. Groups of guinea pigs (*n* = 4) were administrated intramuscularly with M8/M170 (2.5 mg/kg) 1 day before (prophylactic) or after (therapeutic) challenge with 100 ID_50_ of FMDV on the left hind footpad. Guinea pigs injected intramuscularly with PBS before or after challenge were acted as control groups. The day of FMDV infection was marked as day 0. Protection of guinea pigs against FMDV O by passive immunization with M8 or M170 was analyzed in the prophylactic (Fig. 1E, up) and therapeutic (Fig. 1F, up) modes. No lesions on rear feet were considered as full protection. The copies of virus mRNA in the blood sample from guinea pigs of the prophylactic (Fig. 1E, down) and therapeutic (Fig. 1F, down) groups were quantified by the real-time quantitative PCR (RT-qPCR), the limit of detection (LOD) of viral mRNA in blood was labeled. (G) BIAcore SPR kinetics shows the competitive binding of M8/M170 and αvβ6 integrin to FMDV O. For both panels, αvβ6 integrin was immobilized onto the sensor chips. Mixtures of FMDV O (20 nmol/L) with various concentrations of M8 (up) or M170 (down) acted as running phase to flow through the sensor. Binding signals were detected. (H) Amount of virions remaining on the cell surface, as detected by real-time PCR, when exposed to M8 or M170 before or after the virions attach to BHK21 cells. Data is presented as the mean ± SD. Experiments were repeated in triplicate

Given the potent neutralizing activities of M8 and M170 at sub-μmol/L concentrations, we next sought to assess the *in vivo* protection efficacy of these two antibodies in Guinea pigs (GP). Guinea pigs were administered M8 or M170 1 day before or after inoculation of the FMDV, representing a prophylactic (P) or therapeutic (T) setting, respectively (Fig. 1D). In the prophylactic mode, a single intramuscular injection of M8 at 2.5 mg/kg protected 75% of the animals from FMD until the end of the experiments and delayed morbidity in rest of the animals exhibiting symptoms of FMD when compared to the control. In contrast to this, only 25% of the animals receiving the same dose of M170 were protected from FMDV infections, while 75% of the animals developed FMD symptoms later at day 4 PI (Fig. 1E, up). In line with these results, viral loads in the blood were nearly undetectable during the course of the monitoring of the infection in M8 administrated group. However, a severe viremia occurred at day 2 PI in control group. Pre-treatment of M170 alleviated the viremia and cleared ~ 95% of the viral titer when compared to the control group at day 3 PI (Fig. 1E, down). Therapeutic administration of either M8 or M170 one day after virus challenge resulted in a relatively weak protective efficacy with 25% protection for M8 and delayed morbidity for M170 when compared to the control (Fig. 1F, up). Correlated with this, high levels of viral replication prior to therapeutic administration of the NAbs were detected, whereas M8 and M170 treatment at all indicated time-points resulted in largely reduced viral titers with a clear trend for greater reduction with earlier treatment (Fig. 1F, down). Of note, efficient viral infection that had been established at day 1 PI may require a higher dose of antibodies for rapid and complete clearance of FMDV or an earlier therapeutic treatment for suppressing massive replication of viruses.

Integrins, heterodimeric adhesive glycosylated membrane proteins located on the surface of most cells, have been identified as FMDV receptors that bind viruses via a small cleft at the subunit interface of the head (Kotecha et al., 2017). FMDV enters host cells by integrin-mediated endocytosis, then disassociates into pentamers to release RNA in the acidic environment of the endocytic compartments. To investigate whether M8 or M170 interferes with the binding of FMDV to integrin, we performed competitive SPR-based binding assays. The results indicated that both M8 and M170 can prevent FMDV from binding integrin in a concentration-dependent manner (Fig. 1G). Besides, the blocking of binding by M8 appeared more potent when compared to M170, which explains the differential neutralizing activities of these two antibodies (Fig. 1G). Consistent with the competitive binding assay results, in cell-based viral infection model, both M8 and M170 efficiently prevented FMDV type O attachment to the cell surface and could displace the viral particles that had already bound to the cell surface in a dose-dependent manner (Fig. 1H).

To define the key epitopes and atomic determinants of the interactions between FMDV and its two NAbs precisely, we determined the cryo-EM structures of FMDV type O in complex with M8 or M170. Cryo-EM micrographs of the purified formaldehyde-inactivated FMDV mature particles in complex with M8 or M170 were recorded using an FEI Titan Krios electron microscope equipped with a Gatan K2 Summit detector (Fig. S2). Icosahedral reconstructions of FMDV-M8 and FMDV-M170 complexes were achieved at resolutions of 3.2 Å and 3.1 Å, respectively (Fig. S3). The maps for the viral capsid were of sufficient resolution to build and refine the atomic model. But, the densities for antibodies and binding interface were relatively weak due to the unsaturated occupancy and conformational heterogeneity (Fig. S4), which is in line with the structural observations of flexible bindings of integrins to FMDV at different angles (Kotecha et al., 2017). To improve the resolution of the binding interface, local refinement by using an optimized “block-based” reconstruction approach (Wang et al., 2019) was performed. This led to an improvement of the local resolution to 3.9 Å and 3.5 Å for the binding interfaces of FMDV-M8 and FMDV-M170, respectively, enabling a reliable analysis of the interaction modes (Fig. S5).

As expected, M8 and M170 recognize distinct antigenic sites (Fig. 2). M8 binds to the FMDV viral surface within the pentameric building blocks surrounding the fivefold axis at a position akin to the “canyon” in enteroviruses (Dang et al., 2014). In contrast, M170 targets the surface along the edges of pentameric building block of the virus (Fig. 2A–D), nearby the twofold axis at a position similar to that observed for D6 antibody bound to EV71 (Zhu et al., 2018). Apart from targeting distinct antigenic sites, M8 and M170 adopt significantly different configurations upon binding to the FMDV (Fig. S6A). When viewed down the fivefold axis, individual M8 stands vertically, mimicking the association mode between the β subunit of integrin and FMDV (Kotecha et al., 2017), while M170 locates aside M8, adjacent to the twofold axis, but inclines by ~45° towards the viral surface in an attachment mode resembling the strategy used by the α subunit of integrin for its interaction with FMDV (Fig. S6A). However, minor steric clashes resulting from one overlapped residue within the binding sites and the inclined posture of M170 prevent the simultaneous binding of M8 and M170 to FMDV (Fig. S6B), thereby failing to make up a non-competing pair of antibody cocktail.

**Figure 2:**
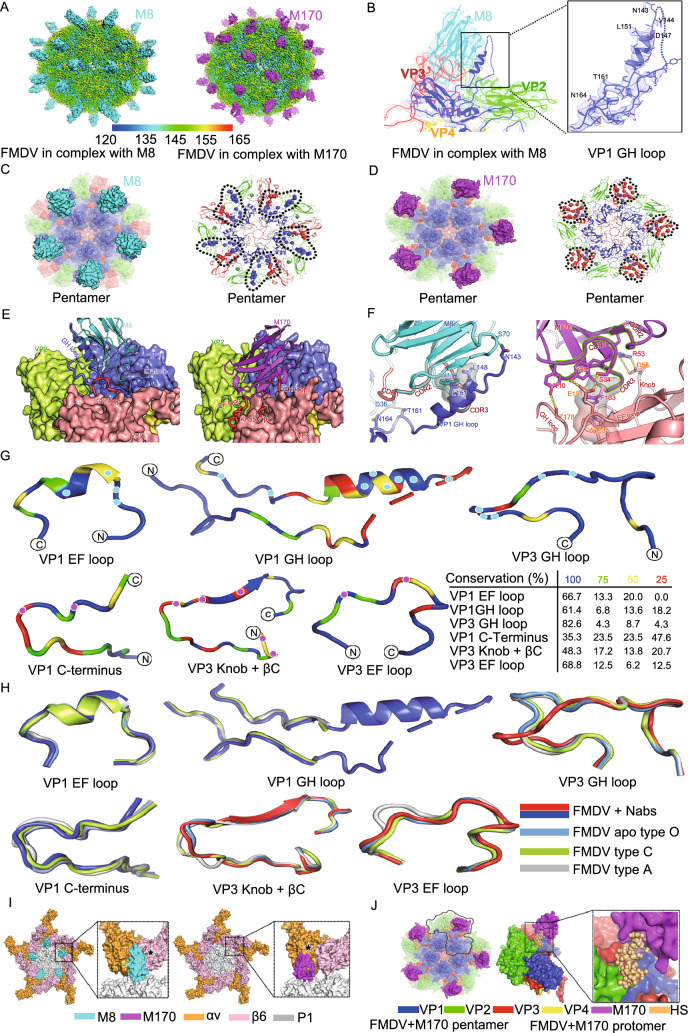
**Cryo-EM structures, mechanism of neutralization of FMDV by M8 or M170**. (A) Cryo-EM maps of FMDV-M8-complex (left) and FMDV-M170-complex (right). The viral capsids of both complexes are rainbow-colored as the color bar shown below; the M8 and M170 are colored in cyan and magenta, respectively. (B) Electron density maps for the binding interface of M8 and one protomer of the FMDV capsid. The inset shows the density maps and atomic model of VP1 GH loop. Residues with side chains are labeled. Surface representation (left) and epitopes (right) of M8 (C) and M170 (D) on a viral pentamer. The pentamers are shown as surface (left) or cartoon (right) in the signature colors (VP1, blue; VP2, green; VP3, red), while M8 and M170 are colored in the same scheme as in Fig. 2A. The epitopes of M8 (right in 2C) and M170 (right in 2D) are shown as spheres and those from one protomeric unit are circled by black dotted lines. (E) The structural basis of M8 (left) and M170 (right) binding to FMDV. Loops from FMDV involved in interactions with M8/M170 are shown as cartoon, while the remaining parts are shown as surface. M8 and M170 are represented as cartoon. The color scheme is as in Fig. 2A. (F) Detailed interactions between FMDV and M8 (left), as well as FMDV and M170 (right). Residues involved in the binding are shown as sticks and hydrogen bonds are shown as yellow dashed lines. The complementary determining regions of M8 and M170 involved in the interaction with viral capsids are outlined in red and green, respectively. (G) Sequence conservation analysis. The loops from FMDV O that are involved in interactions between FMDV and M8 (VP1 GH loop, VP1 EF loop, VP3 GH loop), as well as FMDV and M170 (VP1 C-terminus, VP3 Knob-βC and VP3 EF loop) are aligned with those of 3 other FMDV serotypes (A, Asia 1 and C) and colored according to sequence conservation as listed in the table below. Residues involved in binding to M8 and M170 are marked by cyan and magenta dots. (H) Structural conservation analysis. The same loops in 2C colored in the signature color scheme are superposed with their counterparts from the *apo* O (sky blue), A (gray) and C (lime). (I) Clashes between M8 (left)/M170 (right) and αvβ6 receptor. Superimpositions of structures of the FMDV-M8/M170 and FMDV-αvβ6 are shown in pentamer. The capsid pentamer, M8, M170, and receptor subunits αv and β6 are colored in grey, cyan, magenta, bright orange and light pink, respectively. Clashes between M8/M170 and receptor subunits are prominent and marked with star symbols. (J) Clashes between M170 and HS cellular receptor. Color scheme for capsid pentamer and M170 is same as in Fig. 2D. HS receptor is shown as ball and colored in yellow. A protomer together with its corresponding binders (HS and M170) is marked by black lines. The inset shows the clashes between HS and M170

Analysis of the structures of FMDV in complex with M8 or M170 helped identify the epitopes of these two antibodies. M8 recognizes a conformational epitope constructed by VP1 EF, VP1 GH and VP3 GH loops within a protomer (Figs. 2E and S7). All three complementary determining regions (CDR): CDR1 (residues 32–39), CDR2 (residues 60) and CDR3 (residues 110–119) and framework region (FR, residues 66–70) of M8 are involved in extensive binding contacts with VP1 and VP3, burying ~1,100 Å^2^ of surface area by pinching the helix of VP1 GH loop in the “up” conformation between the CDR2 and CDR3 (Figs. 2B, 2F and S8). Notably, VP1 GH loop, highly disordered in the native virus structures, adopts a more ordered position lying along the viral surface when the disulfide bond linking the base of the loop (C134) to C130 of VP2 is reduced (Logan et al., 1993). The native high-resolution structural details of this loop enable us to define precisely the atomic determinants of the virus-antibody interaction. The epitope recognized by M8 primarily locates in the VP1 EF loop (A93, P94, V95 and L98), VP1 GH loop (K133, N143, V144, D147, L148, Q149, L151, A152, Q153, T161, N164 and A167), and the VP3 GH loop (A171, S172, A175, E176 and T177) (Fig. 2E). When compared to M8, M170 primarily binds to VP3 via recognizing the “knob”, βC, BC, CD, EF and GH loops of VP3, covering most of the exposed surface of VP3, and VP1 C-terminus (Figs. 2E and S7), which is way far beyond the previously reported antigenic site 4 within VP3 (McCahon et al., 1989). The M170 paratope is composed of CDR1 (residues 30–35), CDR2 (residues 53–56) and CDR3 (residues 103–110), which bury a surface area of about ~900 Å^2^ at the interface with VP3 and VP1 through hydrophilic interactions and hydrophobic contacts (Fig. 2E and 2F). The epitope of M170 mainly includes residues 57–59 of the βB “knob”, residues 71 and 73 of BC loop, residue 76 of βC, residues 78 and 85 of CD loop, residues 131 and 134 of EF loop, residues 177, 178 and 183 of GH loop in VP3 and residues 199–202 of VP1 C-terminus (Fig. 2F). Tight binding between the antibodies and FMDV is accomplished by forming extensive hydrophilic interactions, including hydrogen bonds and salt bridges (Tables S2 and S3).

Many NAbs with potent neutralizing activities are virus specific. Cross-reactive NAbs, in general, exhibit relatively weak antiviral activities. By contrast, in our case, M8, capable of cross-binding to several FMDV serotypes, including O, A, Asia 1 and C strains, shows better neutralizing activity than M170 that is a type O-specific antibody (Figs. S1 and 1C). More importantly, cross-reactive NAbs, such as M8, primarily targeting more conserved epitopes offer clues for identifying the Achilles’ heel of viruses between serotypes, even between genera, since these regions can be targeted for the development of antiviral drugs or broad-spectrum vaccine design. To decipher the structural basis for serotype-specific and cross-reactive binding of M8 and M170, sequence and structural alignments of these four FMDV serotypes focusing on key epitopes were performed. As a major antigenic site, VP1 GH loop bears, to some extent, sequence and structural diversity within FMDV serotypes. Therefore, VP1 GH loop together with other antigenic sites can be used to distinguish type O from other serotypes. However, this region also contains a conformationally conserved helix (disordered in the native structures) with a conserved RGD motif within serotypes for receptor binding (Fig. 2G and 2H). Notably, ~70% of the binding interface between M8 and VP1 GH loop is made up by conserved residues among serotypes. Incidentally, VP1 EF and VP3 GH loops exhibit even higher conservation both in sequence and configuration (Fig. 2G and 2H). Interestingly, VP3 GH, rather than other loops, adopts an alternative configuration upon M8 binding, whilst the binding of M170 does not mediate any notable conformational change although the NAb does target VP3 GH loop as well (Fig. S9). Overall, out of the 21 residues in the M8 epitope, 15 (~72%) are identical among these four serotypes (Fig. S7), which explains the cross-reactivity and comparable binding affinities. In contrast to M8, M170 buries ~680 Å^2^ of the VP3 surface by interaction with the “knob”, BC, βC, CD, EF and GH loops as well as ~220 Å^2^ of VP1 via association with its C-terminal loop. Except for VP3 GH loop, subtle conformational variations in the binding areas for M170 are observed within these four serotypes and most residues necessary for M170 binding are not conserved (Fig. 2G and 2H). The specificity of the targeted region both in sequence and configuration explain the serotype-specificity of M170. In addition, residues comprising the epitopes of M170 are highly conserved across currently circulating type O isolates, suggesting that M170 is likely capable of neutralizing most FMDV type O strains (Fig. S10).

In addition to integrin, FMDV can often readily adapt to tissue culture, where infection occurs in the absence of integrin via acquired basic mutations that confer an ability on the virus to interact with heparin sulfate (HS) (SaCarvalho et al., 1997). Cryo-EM structures of FMDV type O in complex with αvβ6, hitherto not at high resolution, reveal that the fully open form of the integrin attaches to an extended GH loop of VP1 via interactions with the RGD motif plus downstream hydrophobic residues and a conserved previously identified HS binding site through an N-linked sugar (Kotecha et al., 2017). Superposition of the FMDV-M8/FMDV-M170 and FMDV-αvβ6 complex structures revealed clashes between the two subunits of αvβ6 and M8/M170 (Fig. 2I). Notably, the structural overlay analysis reveals that M8 competes with β6 subunit for binding to the RGD motif in a similar interaction mode and M170 partially occupies the αv subunit binding site on the virus, where an N-linked sugar from the αv is oriented (Fig. 2I). Thereby, binding of M8 or M170 can completely block the attachment of integrin to FMDV owing to the substantially stronger binding affinities of M8 or M170 for FMDV when compared to that of the integrin. In addition, M170 targets the HS binding site, occluding the infection mediated by sulfated sugars as well (Fig. 2J). Notably, M8 and M170 recognize the RGD motif and the HS binding site, respectively, having great potential to occlude viral mutational escape. Footprints of αvβ6, HS, M8 and M170 on the FMDV surface reveal overlapped patches with areas of ~2,800 Å^2^, ~1,900 Å^2^ and ~120 Å^2^, between αvβ6 and M8/ M170, and between HS and M170, respectively (Figs. S6C and 6D). Collectively, the abilities of M8 and M170 in preventing FMDV from attaching to host cell surface can be attributed to steric clashes arising out of partially overlapping binding sites.

For FMDV, the intact virions with a sedimentation coefficient of 146S can irreversibly dissociate into stable pentamers, referred to as 12S viral capsid degradation products, leading to dramatically decreased immunogenic potency of these vaccines (Paton et al., 2009). It is therefore critical to quantify and continuously monitor the 146S particles present in the crude FMDV antigen preparations used for vaccine manufacturing. A prerequisite to the development of ELISA-based tests for the quality control of FMD vaccines is the availability of antibodies that can specifically detect 146S particles. Antibodies that specifically bind to 146S particles are rarely reported because most of the mAbs can bind to both 146S and 12S particles. In our previous study we reported that M170 specifically interacts with 146S particles, and cannot bind 12S particles directly (Fig. S11) (Harmsen et al., 2011). During the de-assembly of the 146S particle into 12S pentamers, the capsid undergoes significant conformational rearrangements accompanied by a ~4.6º rotation of the bulk of VP3 towards the periphery, giving rise to a structural shift of some distal loops, including EF, HI, DE and BC loops, in the range of 4–9 Å (Fig. S11). Remarkably and in line with the inability of M170 to recognize 12S particles, some residues of the M170 epitope, such as D71 and V73 in BC loop, E131 and K134 in EF loop, lie in the region with the largest conformational changes (Fig. S11). In contrast to M170, M8 shows indistinguishable binding activities against 146S and 12S since its epitope is located beyond this region (Fig. S11). Therefore, the 146S-specific M170, together with 12S-directed antibody, can be combined to develop an ELISA system for the quantification of 146S particles during vaccine manufacturing. The molecular features of the M8 and M170 epitopes unveiled in this study pose interesting targets for structure-based rational broad-spectrum vaccine design and effective antigen detection development for use in vaccine quality control. Our studies also highlight the promise of simultaneous administration of vaccines and therapeutic antibodies for rapid and complete protection prior to the establishment of effective immune responses elicited by FMDV vaccines.

## Electronic supplementary material

Below is the link to the electronic supplementary material.

Electronic supplementary material 1 (PDF 3198 kb)
