# Supplementary material for: Structural and molecular basis for foot-and-mouth disease virus neutralization by two potent protective antibodies
Source: Protein Cell. 2021 Feb 18;13(6):446–53. doi: 10.1007/s13238-021-00828-9 (PMC9095805; doi:10.1007/s13238-021-00828-9)
Supplement: Supplementary file 1 — Electronic supplementary material 1 (PDF 3198 kb) [file 13238_2021_828_MOESM1_ESM.pdf]

Supplemental materials

Materials and methods

Virus production and purification

FMDV serotype O strain O/BY/CHA/2010 (GenBank accession no. JN998085.1) from the OIE/National Foot-and-Mouth Disease Reference Laboratory (Lanzhou, China), was cultured in Baby Hamster Kidney (BHK)-21 cells at 37 ℃ for 8-10 h with Dulbecco's modified Eagle's medium (DMEM) (Gibco, CA, USA) supplemented with 100 U/ml penicillin, 100 μg/ml streptomycin, and 2% fetal bovine serum (FBS, Gibco). After removing the cell debris, the virus supernatant was concentrated using 8% polyethylene glycol (PEG)-6,000 (Sigma-Aldrich, U.S.A). The pellet was resuspended in TNE buffer (50 mM Tris-HCl, 1 mM EDTA, 150 mM NaCl, pH 7.6), and mixed with an equal volume of trichloromethane and then centrifuged at 100,000 g for 1 h. Rude virus solution was loaded onto a 15-45% (w/v) sucrose density gradient, ultra-centrifuged at 120,000 g for 3 h with Beckman SW41 rotor. Target fractions were collected and examined by negative-stain electron microscopy and SDS-PAGE.

Production of M8 and M170 neutralizing antibodies (NAbs)

NAbs (M8 and M170) were isolated and sequenced from phage display immune libraries as previously described by M.M Harmsen (Harmsen et al., 2005). NAb encoding genes were inserted between the *BamH* I and *Xho* I sites of plasmid pGEX-4T-1, and expressed with an N-terminal GST tag in *Escherichia coli*

BL21(DE3) cell. Recombinant proteins were purified by GSTrap HP column (5 ml) on AKTA Pure system (GE), according to the manufacturer′ s instructions. Thrombin (Sigma, USA) was used to cleave the GST tag and an extra step for GSTrap HP column purification was performed to remove cleaved GST tag.

Binding abilities of NAbs to FMDV

The binding abilities of M8 and M170 to FMDV were measured by double antibody sandwich ELISA (Harmsen et al., 2005). Briefly, 96-well plates were coated with 50 ng antibodies (M8, M170, 0.5 μg/ml) per well in coating buffer solution overnight at 4 °C. The plates were filled with 50 μl of 3-fold serially diluted FMDV (initial concentration 3 μg/ml), including O1/BFS1860/UK/67, A TUR/20/2006, A24/Cruzeiro/BRA/55, Asia 1/Shamir/ISR/89, C1/Detmold/FRG/60, and incubated at 25 ℃ for 1 h. The plates were washed for three times and further incubated with biotinylated NAbs (0.25 μg/ml) at 25°C for 1 h. After washing plates, PO-conjugated streptavidin (Jackson Immunoresearch, USA, lot no 79940) was added for another 1 h at 25 °C. Then, the plates were stained with 3,3′,5,5′-tetramethylbenzidine (TMB), and absorbance was measured at 450 nm in a 96-well plate reader.

Plaque-reduction neutralization assay

The plaque-forming unit (PFU) of the viable virus neutralized by NAbs (M8 and M170) was determined using plaque assay. Antibodies were 2-fold serially diluted with the highest concentrations of 51.2 μM, or the cocktail of M8 and M170

(One-third of the neutralizing titer of the antibody plus a 2-fold diluted another antibody), which were incubated with an equal volume of virus (200 PFU/ml) at 37 ℃ for 1 h. The complexes were transferred to a 6-well culture plate with the momolayer BHK21 cells. After further incubating the plate for 1 h with slightly shaking every 20 min, the survival virus attached to the cell surface. The plates were covered with the gum tragacanth (2 ml/well) supplemented with 2% FBS and further incubated for 72 h. After removing the overlay, the cells were washed with PBS and fixed with 4% paraformaldehyde. Plaques were visualized by staining with 2.5% crystal violet. According to the neutralization efficiency of the experimental group to the control sample, the neutralization titers of the two antibodies were calculated by nonlinear curve fitting. The Data is presented as the mean ± SD of triplicate measurements.

Binding affinity measurements

SPR experiments were performed by a BIAcore 8k machine with CM5 sensor chips (both GE Healthcare) in PBST buffer (PBS, 0.05% Tween-20(v/v)). Due to the requirement of acidic condition for the sensor-labeling, NAbs or receptors were loaded onto the sensor. The serially diluted FMDV (0, 2.5, 5, 10, 20 and 40 nM) flowed over the NAb (M8 or M170)-immobilized CM5 sensor chip surface. The binding affinities of two NAbs were analyzed using the software BIAevaluation Version 4.1. To further analyze the ability of two NAbs simultaneously to bind the virus, inactivated FMDV viruses firstly flowed through one NAb-immobilized CM5

chip. Prior to the other NAb flowing through, the same NAb acted as the flow phase to fully occupy the binding sites on virus surface, and then the other NAb flowed through for binding signal detecting. For blocking virus-receptor interaction, αvβ6 integrin receptor (R&D, lot: DCHM0319011) was immobilized on CM5 chips at concentrations equivalent to ~250 response units. Mixtures of 20 nM FMDV and different concentrations of NAbs flowed over the chip. Binding signals were detected and analyzed with the software BIAevaluation Version 4.1.

FMDV challenge in guinea pigs

Guinea pigs (weight 300-400 g) were randomly divided into six groups, including therapeutic and prophylactic groups. Briefly, groups of guinea pigs were administrated intramuscularly with M8/M170 (2.5 mg/kg) 1 day before (prophylactic) or after (therapeutic) challenge with 100 50% median infective doses (100 ID_{50}) of FMDV (0.2 ml) on the left hind footpad. Guinea pigs injected intramuscularly with PBS before or after challenge were acted as control groups. All animals were examined for clinical symptoms at 1 to 10 days post-infection (DPI). No lesions were considered as full protection, and blood samples were collected for detecting the viral RNA copies by real-time quantitative PCR (RT-qPCR). In brief, the total RNA of samples was extracted by TRIzol reagent (Invitrogen) for the synthesis of cDNA using PrimeScript ™ RT Master Mix (TaKaRa, Dalian, China). RT-qPCR was performed on a CFX96 Touch™ Real-Time PCR Detection System (Bio-Rad Laboratories, Hercules, CA, USA), by 40 cycles of denaturation at 95 ℃ for 30 s,

annealing and extension at 60 °C for 30 s. A total of 20 µl reaction system contains 10 µl 2X Premix Ex Taq II (TaKaRa, Dalian, China), 1 µl of 3D gene specific primer (forward: 5' ACTGGGTTTTACAAACCTGTG A 3'; reverse: 5' GCGAGTCCTGCCACGGA 3'), 2 µl of fluorescent probe (5' TCC TTT GCA CGC CGT GGG AC 3'), and 2 µl of the cDNA template. The $pcDNA_{3.1}$-3D plasmids was constructed and quantified as a standard sample.

Virus quantification on the cell surface by RT-PCR

The amounts of FMDV remaining on the surface of BHK21 cells after M8/M170 treatment were estimated using qPCR as previously described. Briefly, FMDV was incubated with the serially diluted NAbs before and after the virus attached to BHK21 cells (MOI=1) at 4 ℃. The cells were washed three times and the total RNA was extracted by TRIzol reagent (Invitrogen). The cDNA was prepared by PrimeScript ™ RT Master Mix (TaKaRa, Dalian, China). The level of virus mRNA was quantified using SYBR Premix Ex Tag II (Tli RnaseH Plus) on CFX96 Touch™ Real-Time PCR Detection System (Bio-Rad Laboratories, Hercules, CA, USA), the reaction system is the same as before. The level of glyceraldehyde-3-phosphate dehydrogenase (GAPDH forward: 5' AAGAAGGTGGTGAAGCAGGCATC 3', GAPDH reverse: 5' CGCCATCGAAGGTGGAAGAGTG 3') was used as an internal control. The relative levels of mRNA in different samples were represented using the $2^{-\Delta\Delta ct}$ method (Livak and Schmittgen, 2001).

Thermofluor Assay

Thermofluor assay was performed with a MX3005p RT-PCR instrument (Agilent), SYTO9 (invitrogen) was used as fluorescent probe to detect the single-stranded RNA from virus capsid. In brief, the 50 µl reaction system includes, 2 µg purified viruses or 2 µg of viruses plus 1.5 µg of NAbs (~120 antibody molecules per FMDV virion), or 37 ℃ treated viruses or virus-antibody complexes and 5 µM SYTO9 in PBS buffer solution. System program was ramped from 25 to 99 ℃ with fluorescence recorded in triplicate at 1 ℃ intervals.

Cryo-EM and data collection

Purified M8/M170 were incubated with purified FMDV particles (at a concentration of 0.5 mg/ml) at 4 °C for 1 min at the ratio of ~300 NAbs per FMDV particle. A 3 µl aliquot of the complex of FMDV and M8/M170 were applied to a freshly glow-discharged 400-mesh holey carbon-coated copper grid (C-flat, CF-2/1-2C, Protochips). Grids were blotted for 3 s in 90% relative humidity for plunge-freezing (Vitrobot; FEI) in liquid ethane. Cryo-EM datasets of FMDV-M8 and FMDV-M170 were collected with Talos Arctica and Titan Krios microscopes (FEI), both of which were equipped with a direct electron detector (K2 Summit; Gatan). Movies ((25 frames, each 0.2 s, total dose 30 e^{-} $Å^{-2}$) were recorded with a defocus between 1.2 and 2.8 µm. Automated single-particle data acquisition was performed by SerialEM (Mastronarde, 2005), with a calibrated magnification of 59,000 yielding a final pixel size of 1.32 Å and 1.35 Å for FMDV-M8 and FMDV-M170, respectively.

Image processing, model building and refinement

A total of 2,493 micrographs (FMDV-M8 complex) and 298 micrographs (FMDV-M170 complex) were recorded, respectively. Frames were corrected for beam-induced draft by aligning and averaging the individual frame of each movie using MOTIONCORR2 (Li et al., 2013). The contrast transfer function parameters were estimated by Gctf (Zhang, 2016). Particles were picked manually by Manual pick in RELION3 (Scheres, 2012). A total of 5,508 particles and 3,800 particles for FMDV-M8 and FMDV-M170 complexes were picked, respectively and selected to two-dimensional alignment and three-dimensional reconstruction. Finally, 3,701 particles of FDMV-M8 complex and 3,692 particles of FMDV-M170 complex were used for the icosahedral symmetry reconstruction. The resolution of the final icosahedral reconstructions was 3.2 Å and 3.1 Å, as evaluated by Fourier shell correction (threshold = 0.143 criterion). Although the overall resolution for these two icosahedral reconstructions is up to 3.1 Å – 3.2 Å, the maps for the binding interface between FMDV and NAbs are quite weak due to the relative low occupancy of NAbs and conformational heterogeneity. To improve the resolution for the binding interface, we used the block-based reconstruction strategy for focusing classification and refinement. The orientation parameters of each particle determined in Relion were used to guide extraction of the block region (~50% bigger than protomer-NAb) and these blocks were further 3D classified. A local reconstruction focusing on the protomer-NAb region was carried out, yielding a resolution of 3.9 Å and 3.5 Å for the

interface of FMDV-M8 and FMDV-M170, respectively. The atomic model of FMDV (PDB code: 5DDJ) was initially fitted into our maps with CHIMERA (Pettersen et al., 2004) and future corrected manually by real-space refinement in COOT (Emsley and Cowtan, 2004). The atomic models of M8 and M170 were built *de novo* into densities with structures of single-domain antibodies as a guide, using COOT. These models were further refined by positional and B-factor refinement in real space with Phenix (Afonine et al., 2012). Refinement statistics are summarized in Table S1.

Reference

Afonine, P.V., Grosse-Kunstleve, R.W., Echols, N., Headd, J.J., Moriarty, N.W., Mustyakimov, M., Terwilliger, T.C., Urzhumtsev, A., Zwart, P.H., and Adams, P.D. (2012). Towards automated crystallographic structure refinement with phenix.refine. Acta crystallographica Section D, Biological crystallography 68, 352-367.

Emsley, P., and Cowtan, K. (2004). Coot: model-building tools for molecular graphics. Acta crystallographica Section D, Biological crystallography 60, 2126-2132.

Harmsen, M.M., Van Solt, C.B., Fijten, H.P., and Van Setten, M.C. (2005). Prolonged in vivo residence times of llama single-domain antibody fragments in pigs by binding to porcine immunoglobulins. Vaccine 23, 4926-4934.

Li, X., Mooney, P., Zheng, S., Booth, C.R., Braunfeld, M.B., Gubbens, S., Agard, D.A., and Cheng, Y. (2013). Electron counting and beam-induced motion correction enable near-atomic-resolution single-particle cryo-EM. Nature methods 10, 584-590.

Livak, K.J., and Schmittgen, T.D. (2001). Analysis of relative gene expression data using real-time quantitative PCR and the 2(-Delta Delta C(T)) Method. Methods 25, 402-408.

Mastronarde, D.N. (2005). Automated electron microscope tomography using robust prediction of specimen movements. Journal of structural biology 152, 36-51.

Pettersen, E.F., Goddard, T.D., Huang, C.C., Couch, G.S., Greenblatt, D.M., Meng, E.C., and Ferrin, T.E. (2004). UCSF Chimera--a visualization system for exploratory research and analysis. Journal of computational chemistry 25, 1605-1612.

Scheres, S.H. (2012). RELION: implementation of a Bayesian approach to cryo-EM structure determination. Journal of structural biology 180, 519-530.

Zhang, K. (2016). Gctf: Real-time CTF determination and correction. Journal of structural biology 193, 1-12.

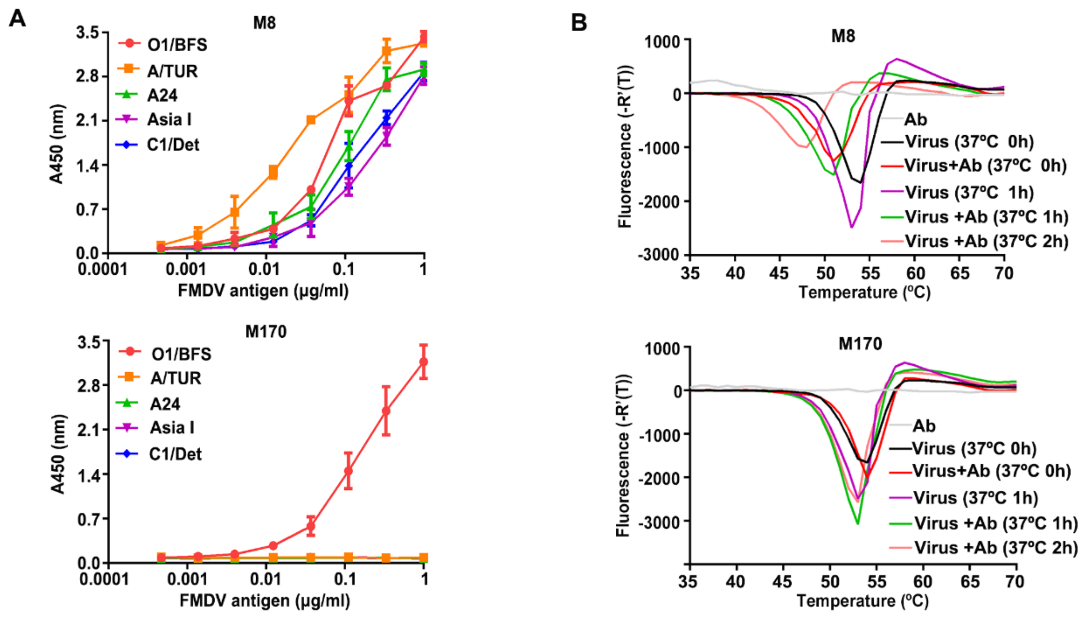

Supplementary Figure 1. Stability and binding analysis of M8 and M170 against FMDV. (A) Dose-dependent binding analysis of M8 and M170 against representative FMDV serotypes by ELISA. Each plot represents the mean of OD450 values from triplicate wells. Error bars represent mean ± SD. FMDV O (O1/BFS1860/UK/67), A (A/TUR/20/2006, A24/Cruzeiro/BRA/55), Asia 1 (Asia 1/Shamir/ISR/89) and C (C1/Detmold/FRG/60). (B) Stabilities of FMDV O upon addition of M8 (top) or M170 (bottom) at physiological temperature. The release of RNA reflecting the dissociation of capsids was detected by an increase in the fluorescence signal (SYTO9 fluorescent).

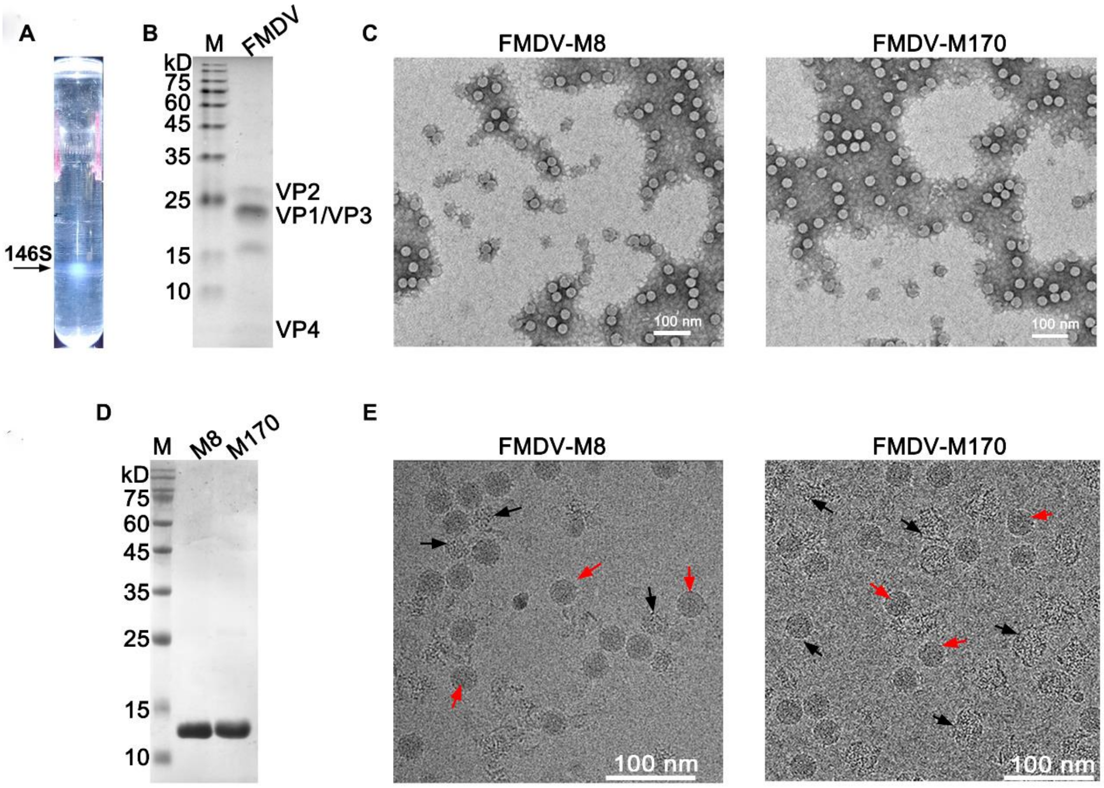

Supplementary Figure 2. Purification and characterization of M8, M170 and FMDV. (A) Zonal ultracentrifugation of a 15 to 45% (w/v) sucrose density gradient at 120,000 g for 3 h was used to purify FMDV from the harvest concentrate described in the method section. Only one type of FMDV particle, corresponding to the 146S mature virion, was separated. (B) SDS-PAGE analysis for FMDV capsid proteins, the theoretical molecular weights of VP1, VP2, VP3 and VP4 are 23.7 kDa, 24.4 kDa, 23.9 kDa and 8.9 kDa, respectively. (C) The negative-stain images of FMDV in complex with M8 and FMDV in complex with M170. (D) Purity evaluation of M8 and M170 by SDS-PAGE. (E) The cryo-EM micrographs of FMDV-M8 and FMDV-M170 complexes, and the intact particles and broken particles were marked by red and black arrows, respectively.

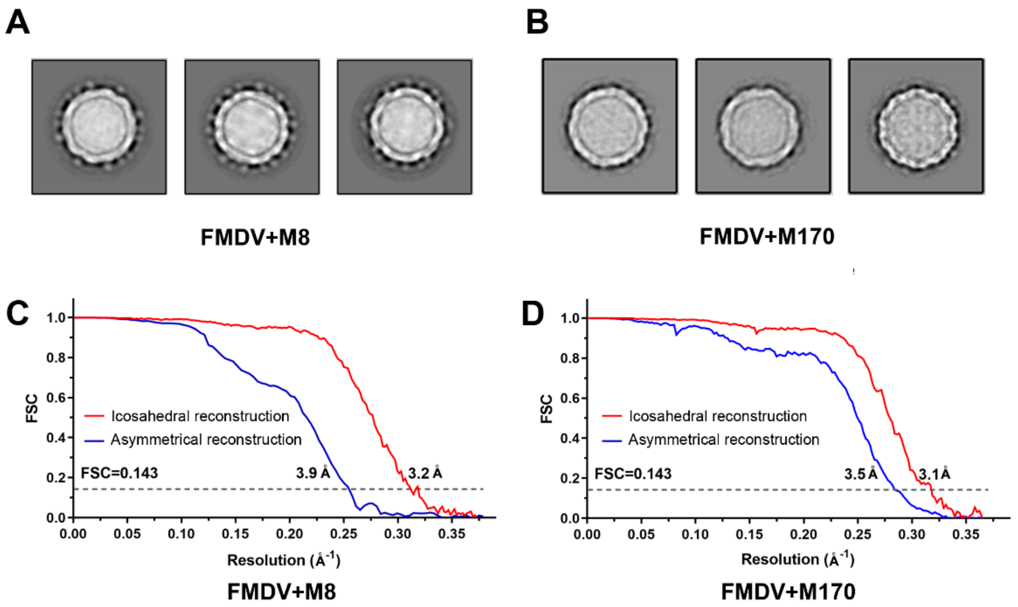

Supplementary Figure 3. 2D classification and FSC curves. Representative classes from 2D classification in RELION for FMDV-M8 (A) and FMDV-M170 complexes (B). Gold-standard Fourier shell correlation (FSC) curves of the final maps of FMDV-M8 (C) and FMDV-M170 complexes (D).

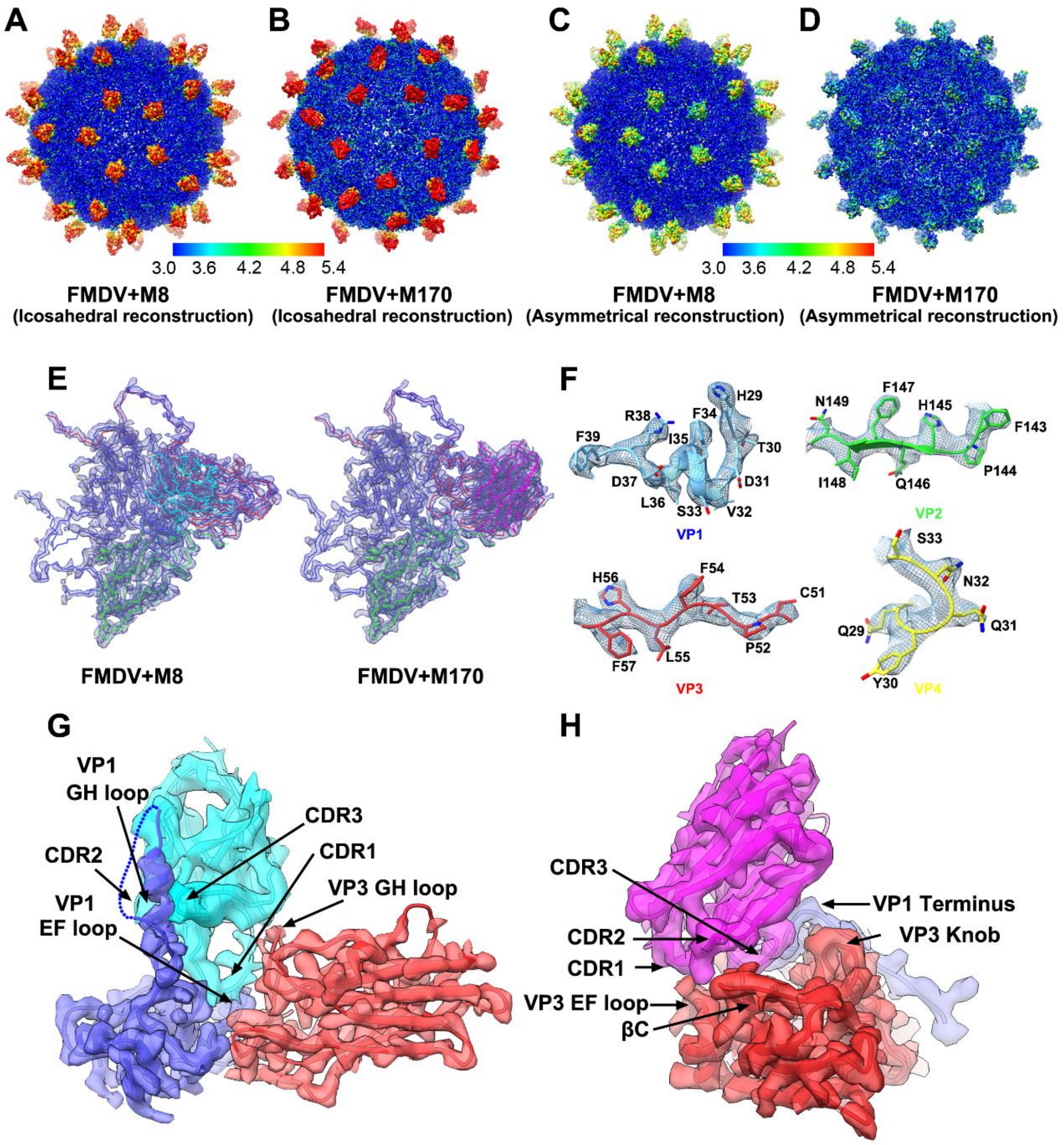

Supplementary Figure 4. Map resolution evaluation and electron density maps. Map resolution assessment of the icosahedral reconstructions of FMDV-M8 (A) and FMDV-M170 (B), and the asymmetrical reconstructions of FMDV-M8 (C) and FMDV-M170 (D) with color indicated below. Electron density maps for the FMDV protomer (E), the sidechains of VP1-VP4 (F) and the binding interface of M8 (G)/M170 (H) are shown.

A

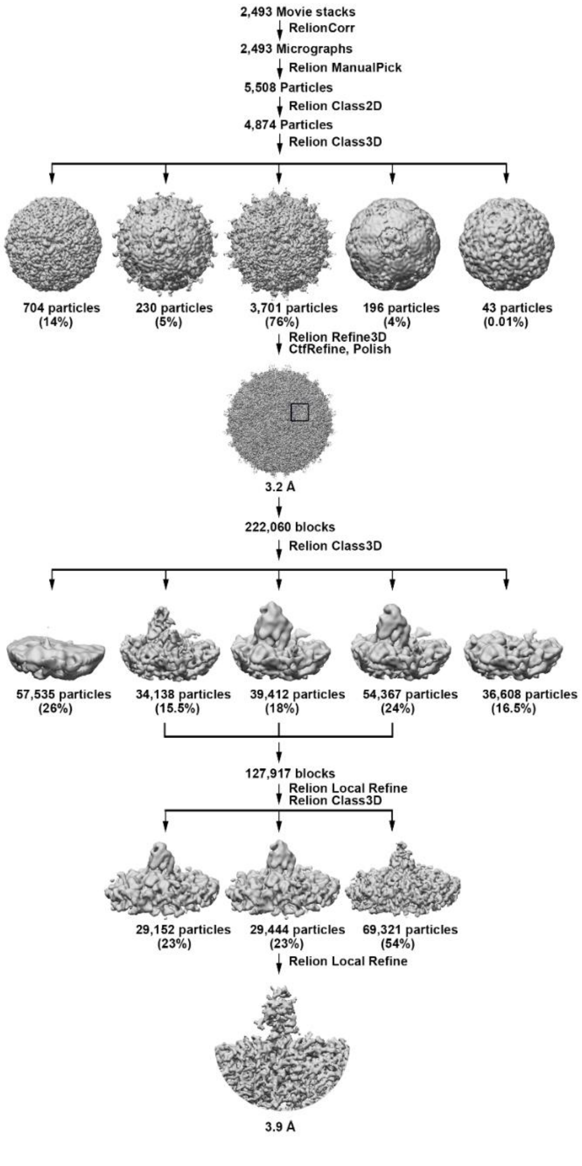

2,493 Movie stacks
RelionCorr
2,493 Micrographs
Relion ManualPick
5,508 Particles
Relion Class2D
4,874 Particles
Relion Class3D
704 particles (14%)
230 particles (5%)
3,701 particles (76%)
196 particles (4%)
43 particles (0.01%)
Relion Refine3D
CtfRefine, Polish
3.2 Å
222,060 blocks
Relion Class3D
57,535 particles (26%)
34,138 particles (15.5%)
39,412 particles (18%)
54,367 particles (24%)
36,608 particles (16.5%)
127,917 blocks
Relion Local Refine
Relion Class3D
29,152 particles (23%)
29,444 particles (23%)
69,321 particles (54%)
Relion Local Refine
3.9 Å

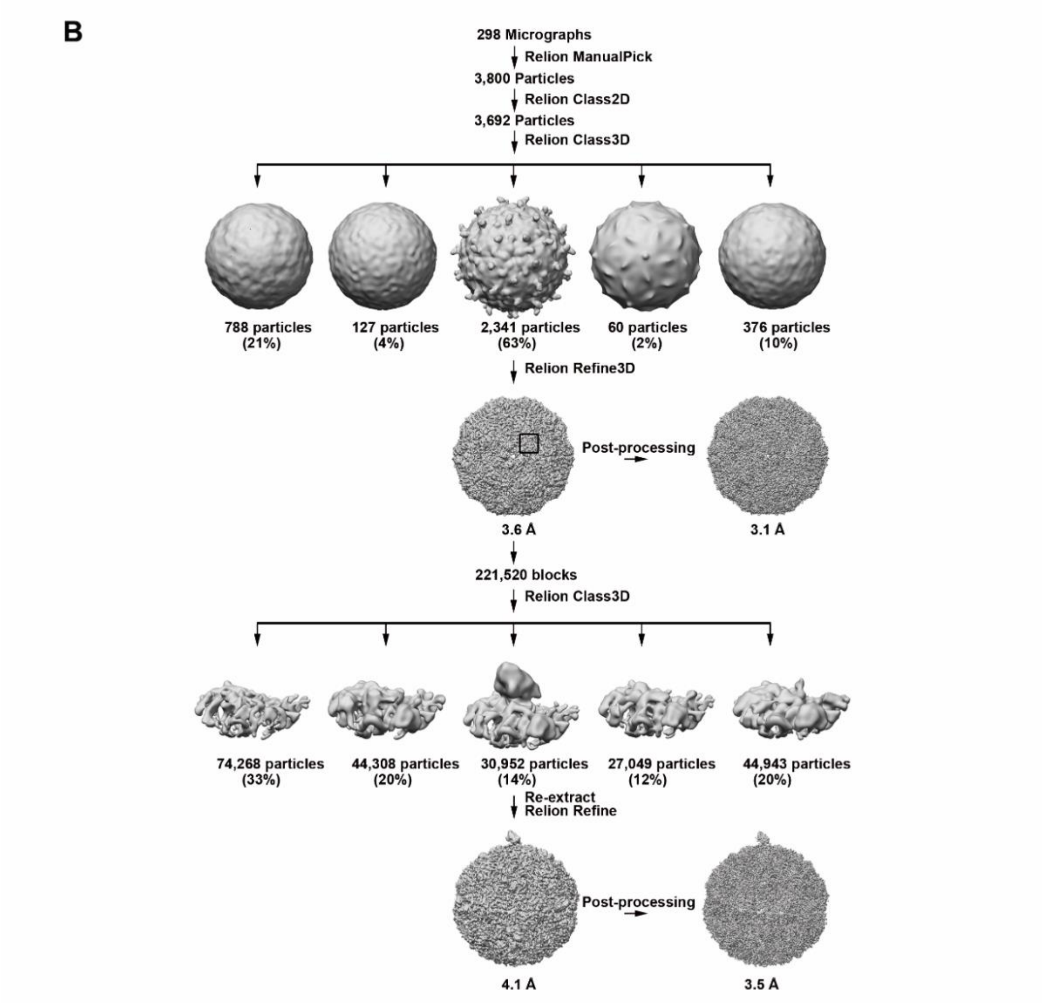

Supplementary Figure 5. Flow-chart for Cryo-EM data processing. (A) and (B) show the data processing procedures for FMDV-M8 and FMDV-M170 complexes, respectively. Details can be found in the Methods section.

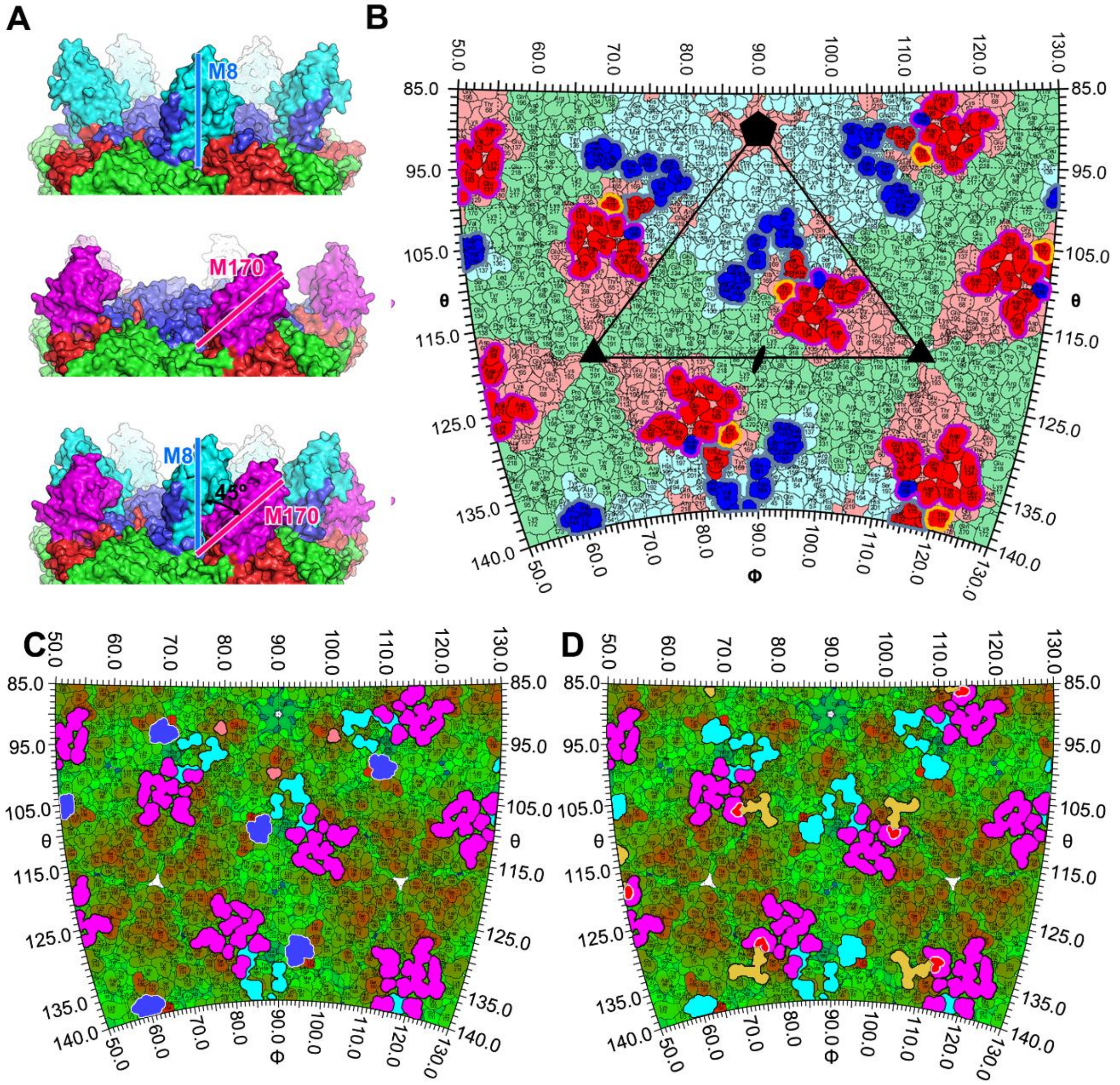

Supplementary Figure 6. The NAb (M8 and M170) and receptor (αvβ6 and HS) footprints on the viral surface. (A) Side views of two NAbs bound to a pentamer. up: FMDV-M8 complex, and middle: FMDV-M170 complex, down: the superimposition of two complexes shows an angle of ~45 ° between M8 and M170. The same color scheme is applied as above. (B) The M8 and M170 footprints on the FMDV surface. Residues of VP1, VP2, and VP3 are outlined in blue, green, and red, respectively. Residues involved in binding to NAbs are shown in brighter colors corresponding to the protein chain they belong to, the footprints of M8 and M170 are indicated by gray and magenta lines, respectively. The overlapped residue for binding to both M8 and

M170 is marked by yellow lines. (C) and (D) Roadmap showing the relative positions of the NAb (M8 and M170) and receptor (αvβ6 and HS) footprints on the viral surface. The footprints of M8, M170, αvβ6 and HS receptor are outlined with cyan, magenta, light pink and yellow line, respectively. Overlapped footprints between M8 and αvβ6 and between M170 and HS are highlighted in blue and red.

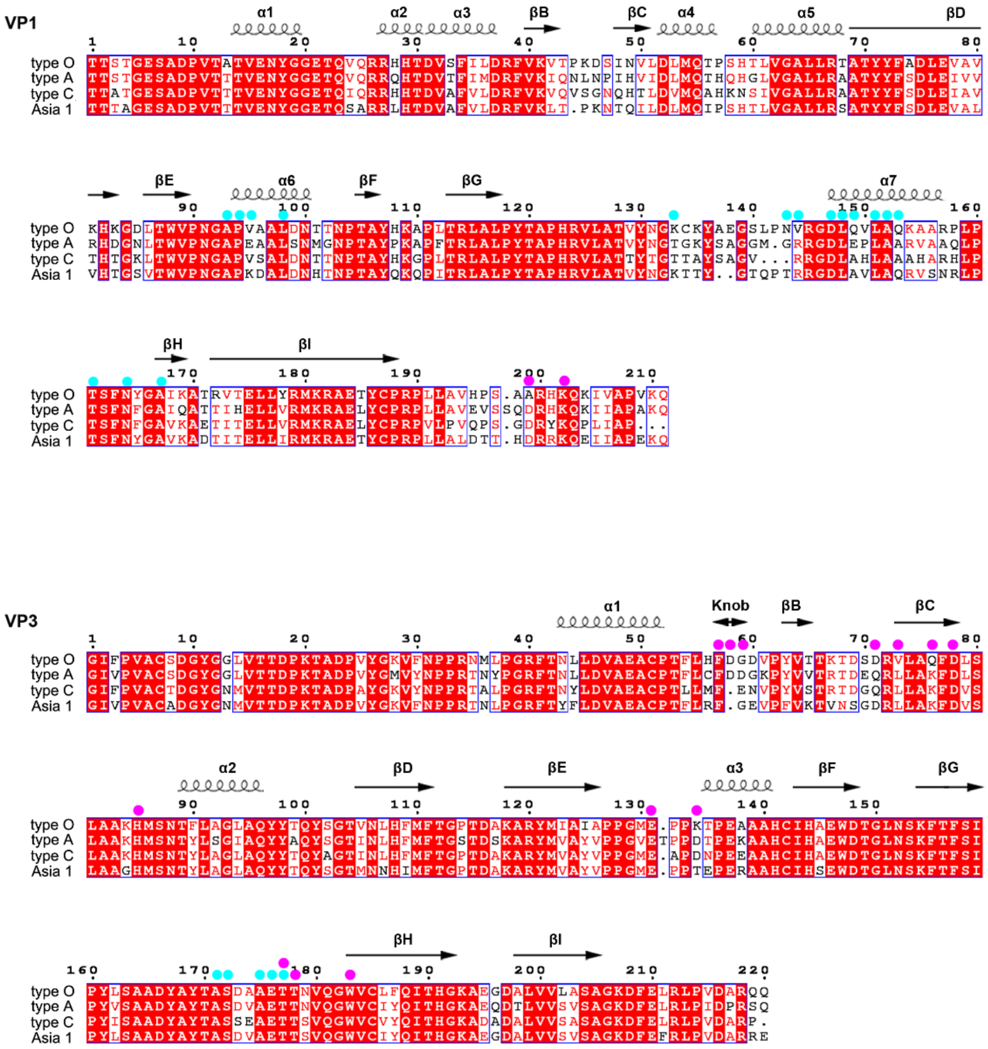

Supplementary Figure 7. Sequence alignment of capsids between the four representative FMDV serotypes. Sequence alignment of VP1 and VP3 from FMDV O with counterparts from 3 representative FMDV serotypes (A, C and Asia I). The residues involved in directly interacting with M8 and M170 are marked with cyan and magenta balls, respectively.

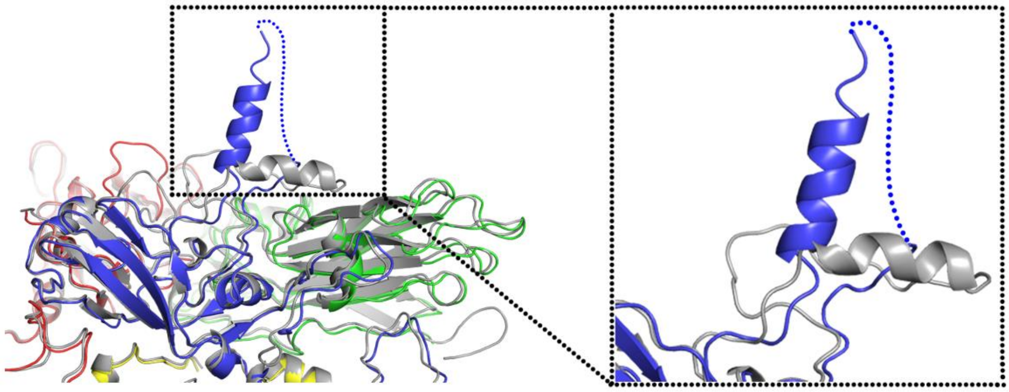

Supplementary Figure 8. Conformational comparison of the VP1 GH loop. The VP1 GH loop in the structure of FMDV-M8 complex exhibits an “up” configuration compared to the “down” conformation in the structure of the reductant treated FMDV (PDB Code: 1FOD). The color scheme for VP1-VP4 in the structure of FMDV-M8 complex is same as the Fig. 2B and the protomer from the reductant treated FMDV is colored in gray.

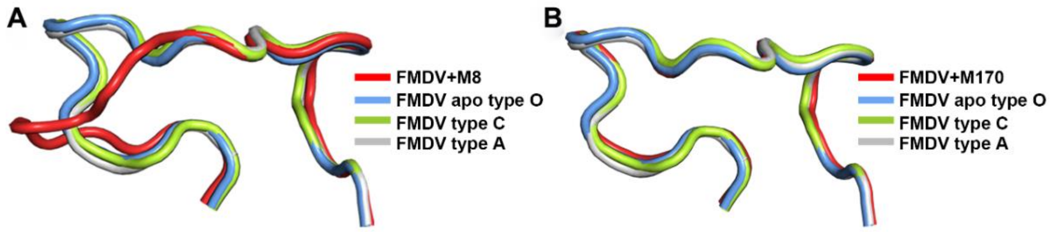

Supplementary Figure 9. Conformation conservation analysis of the VP3 GH loop. Structures of the VP3 GH loop from FMDV-M8 (A) and FMDV-M170 (B) are superposed with counterparts from 3 representative FMDV serotypes.

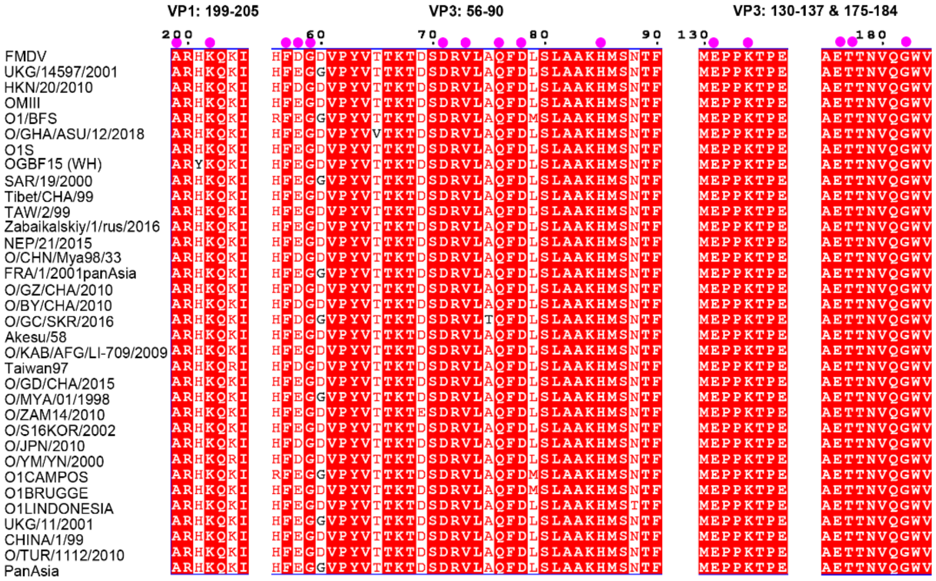

Supplementary Figure 10. Multiple-sequence alignment analysis of the M170 epitope on FMDV O. The alignment results are displayed with the program Espript.

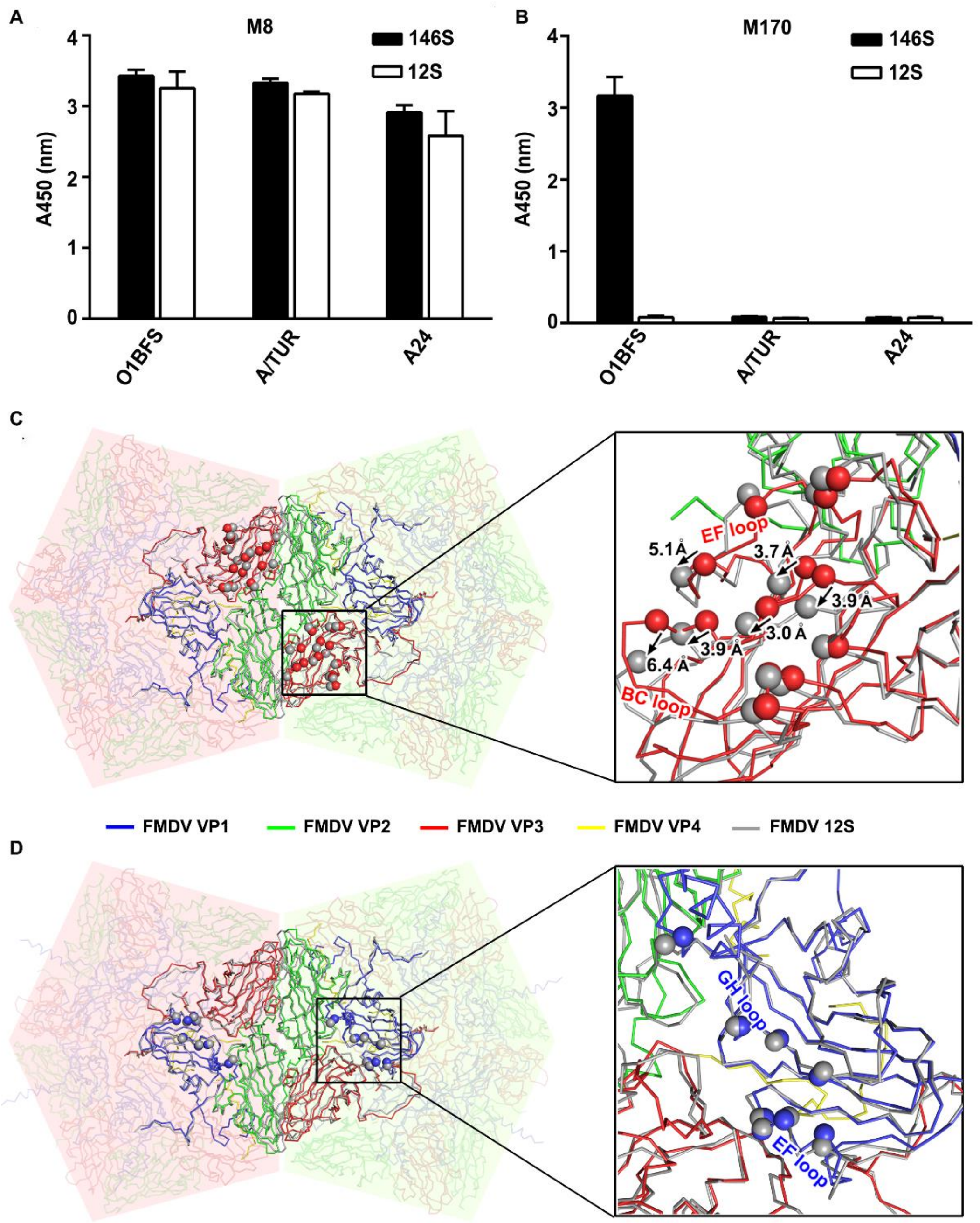

Supplementary Figure 11. Structural basis of binding preference/specificity of M8/M170 for 146S or 12S particles. (A) M8 exhibited no clear binding preference for 146S or 12S particles. (B) M170 showed specifically binding to 146S particles. Purified viruses (146S) and acid-dissociated pentamers (12S) from FMDV types A and O were used for testing the binding to M8 and M170 by ELISA. Structural analysis for explaining the binding specificity of M170 for 146S particles (C) and no

binding preference of M8 for 146S or 12S particles (D). Superimposition of structures of the pentamers from 146S and 12S (PDB:5OYI) shows structural rearrangement in VP3, in particular the distal loops, such as BC and EF loops. Color scheme for the pentamer from 146S is same as Fig. 2B and the structures of acid-dissociated pentamers are colored in gray. Epitopes of M8 and M170 are represented as spheres and colored corresponding to the protein chain they belong to. Major structural shifts are labeled and marked by black arrows.

Supplementary Table 1 Cryo-EM data collection and atomic models refinement statistics

Data collection

Complex	FMDV+M8	FMDV+M170
Microscope	FEI Talos Arctica	FEI Titan Krios
Camera	Gatan K2	Gatan K2
Voltage (kV)	200	300
Total dose (e^-/A^2)	30	30
Micrographs (total)	2,493	298
Micrographs (used)	2,493	298
Particles selected	5,508	3,800
Particles included in final reconstruction	3,701	3,692
sampling, Å per pixel	1.32	1.35
Defocus range (µm)	1.2-2.8	1.2-2.8
Symmetry	I	I
Resolution (Å) (FSC=0.143 criterion)	3.2	3.1
Block particles included in final reconstruction	29,444	30,952
Symmetry imposed on block particles	C1	C1
Resolution (Å) (C1 reconstruction)	3.9	3.5

Model refinement

Ramachandran statistics (%)		
Most favored	92.88	96.07
Allowed	6.36	3.93
Outliers	0.76	0
R.m.s.d		
Bond lengths (Å)	0.014	0.008
Bond angles (°)	1.228	0.757

Supplementary Table 2. Residues of M8 interacting with the FMDV (d < 4 Å)

FMDV			M8
**Location**	**Domain**	**Residues**	**Residues**
VP1	EF loop	A93	I34 N35
		P94	S33 I34
		V95	F32 S33 I34 N35
		L98	N35
	GH loop	K133	N110
		N143	D69 S70
		V144	Y67 D69
		D147	A66
		L148	A66 Y67 W113
		Q149	A111
		L151	T60 A66 W113
		A152	N110 A111 W113
		Q153	A111
		T161	D36 I39
		N164	D36
		A167	N35
VP3	GH loop	A171	I34
		S172	S33 I34
		A175	G118 T119
		E176	F117
		T177	F117

Supplementary Table 3. Residues of M170 interacting with the FMDV (d < 4 Å)

FMDV			M170
**Location**	**Domain**	**Residues**	**Residues**
**VP1**	**C-terminus**	A199	A104 P106 S108
		K202	S108
**VP3**	**Knob**	F57	L105
		D58	R53 L105
		G59	Y62
	**BC loop**	D71	W56
		V73	W56
	**βC**	Q76	A104 L105
	**CD loop**	D78	F103
		H85	L105
	**EF loop**	E131	R30 S34 Y35
		K134	W56
	**GH loop**	T177	D109 Y110
		T178	Y110
		W183	F103
